# *In vivo* fate mapping of cryopreserved murine ovarian grafts

**DOI:** 10.1186/s13048-014-0081-7

**Published:** 2014-08-15

**Authors:** Chi-Huang Chen, Shun-Jen Tan, Chii-Ruey Tzeng

**Affiliations:** Department of Obstetrics and Gynecology, School of Medicine, College of Medicine, Taipei Medical University, Taipei, Taiwan; Center for Reproductive Medicine, Department of Obstetrics and Gynecology, Taipei Medical University Hospital, Taipei, Taiwan; Department of Obstetrics and Gynecology, Tri-Service General Hospital, National Defense Medical Center, Taipei, Taiwan; Graduate Institute of Clinical Medicine, College of Medicine, Taipei Medical University, Taipei, Taiwan

**Keywords:** AMH, Bioluminescence imaging, Cryopreservation, Ovary, Transgenic

## Abstract

**Background:**

Cryopreservation of ovarian tissue has been suggested as an alternative to restore fertility for ovarian failure before chemotherapy.

**Methods:**

Ovaries of donor FVB/N-Tg (*Pol*II–Luc) Ltc transgenic mice (n = 5) were cryopreserved and transplanted to the back muscles of recipient FVB/NJNarl wild-type mice that had undergone bilateral oophorectomy. We evaluated the fate of cryopreserved murine ovarian grafts by *in vivo* bioluminescent imaging (BLI), AMH mRNA expression and follicle counts.

**Results:**

There were significantly stronger BLI signals in the fresh ovaries than in the frozen–thawed ones. The number of primordial follicles was significantly lower in frozen–thawed ovaries at 10 days after transplantation (P < 0.001). The AMH mRNA expression was significantly lower in the frozen–thawed ovaries (P < 0.001), showing that unavoidable harm occurs after transplantation.

**Conclusions:**

Ovarian cryopreservation by slow freezing compromises ovarian reserve by cryoinjury and ischemia, evident at an early stage after transplantation.

## Background

Cryopreservation of ovarian tissue has been conducted since the 1990s and has been suggested as an alternative to restore fertility for girls and women who are at high risk for ovarian failure after chemotherapy or radiotherapy [1–3]. This technique has several advantages over the simple cryopreservation of oocytes and embryos. First, in theory, it should preserve a large number of immature oocytes within primordial follicles, independent of the menstrual cycle, and does not delay cancer therapy. Second, cryopreservation of ovarian tissue can preserve the endocrine functions of the ovary, which cannot be achieved by cryopreserving the oocyte or embryo. In addition, this strategy is also suitable for prepubertal girls [4,5]. Cryopreservation of ovarian tissues has generally been performed using slow freezing methods using a programmed freezer [6,7]. However, cryopreservation of ovarian tissue is complicated, as it is comprises several different cell types that have different requirements for survival.

Primordial follicles have been reported to have tolerance to cryopreservation because of their relative abundance (90% of the ovary by mass), relatively inactive metabolic rate, small size and absence of the zona pellucida and peripheral cortical granules [8–10]. However, the effect of cryopreservation of ovarian tissue on the development of primordial follicles remains to be elucidated. Anti-Müllerian hormone (AMH) is expressed in granulosa cells and is considered to be the best hormonal marker for the ovarian follicular reserve. Thus, a measure of AMH can be applied to the clinical evaluation of follicular reserves and function [11–14].

With the advent of *in vivo* bioluminescent imaging (BLI), luciferase reporter genes have been used widely in various biomedical fields for the noninvasive investigation of cellular and molecular events involved in normal and pathological processes [15]. In this study, our objective is to evaluate the fate of cryopreserved murine ovarian grafts by using *in vivo* BLI in real time, based on longitudinal tracking of the individual subjects quantitatively in the short term, and by measuring the expression of AMH mRNA using real-time quantitative polymerase chain reaction (qPCR) amplification and follicle counts as the evidence whether the ovarian cryopreservation by slow freezing method was compromised the ovarian reserve at an early stage after transplantation.

## Methods

### Mice

Five-week-old FVB/N-Tg (*PolII-luc*) Ltc transgenic female mice with an H-2 haplotype (H_2_^q^) were used as ovarian tissue donors. The gene fragment construct *polII-luc* encodes a 712-bp mouse RNA polymerase (Pol) II promoter and a modified firefly luciferase cDNA. This transgenic line was created using microinjection of the *polII-Luc* transgene into fertilized FVB/N embryo pronuclei and maintained as a hemizygotic stock (Level Transgenic Center of Level Biotechnology Inc. Taipei, Taiwan). Age-matched inbred FVB/NJNarl wild-type mice (National Laboratory Animal Center, Taipei, Taiwan) were used as recipients (n = 5). All mice were housed under a 12 h light: 12 h dark regimen at 22–24°C, with food and water ad libitum. All procedures were reviewed and approved by the Animal Experimental Committee at the National Defense Medical Center and Tri-Service General Hospital, Taipei, Taiwan, in accordance with the Guiding Principles for the Care and Use of Laboratory Animals.

### Study design

**Figure 1:**
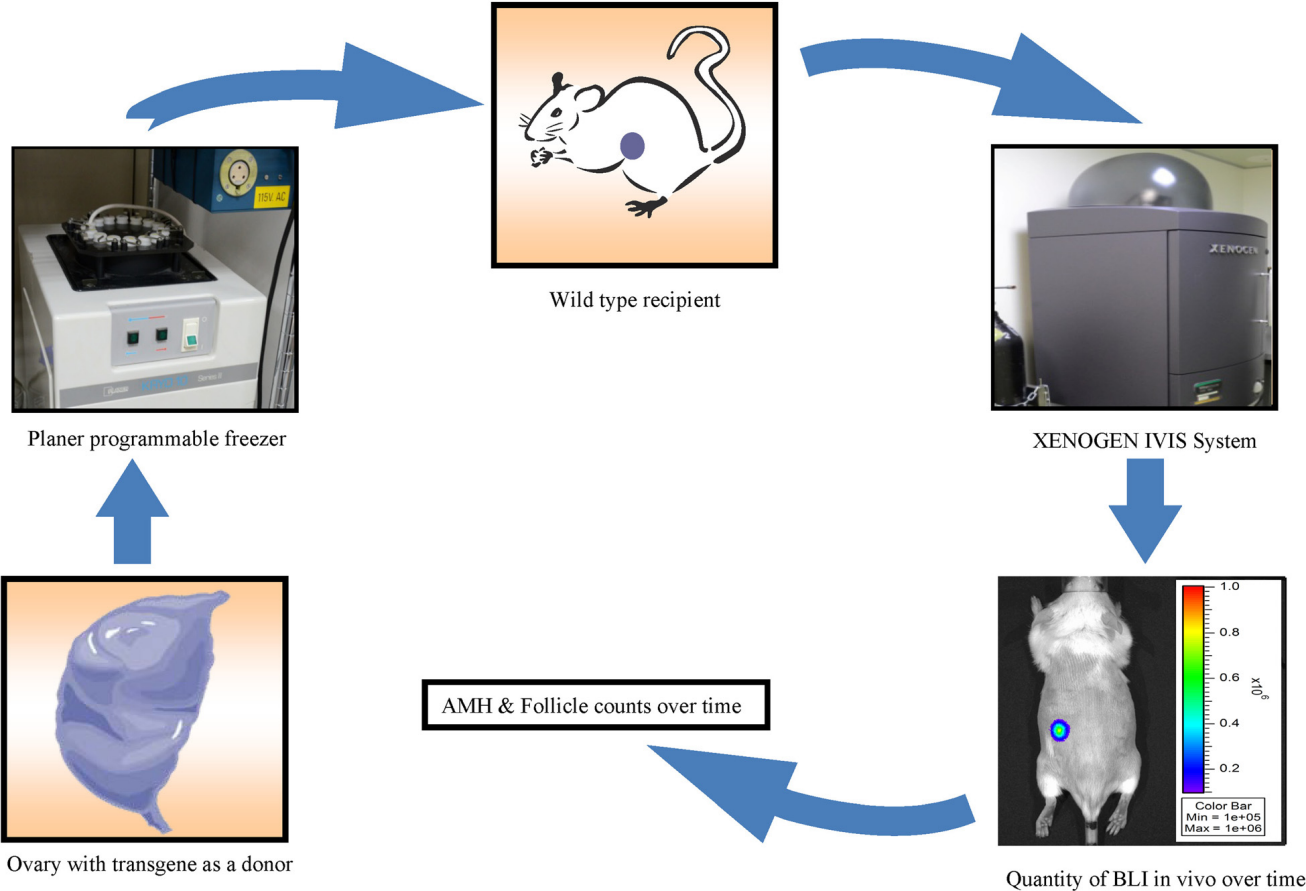
**Scheme of the study design.**

The mice were treated intraperitoneally (i.p.) with ketamine (50 mg/kg) and xylazine (15 mg/kg) to induce general anesthesia. Recipient mice underwent bilateral ovariectomy 1 week before transplantation. Transgenic fresh or frozen–thawed ovarian tissues were transplanted under the back muscle of the donor animals (Figure 1). Recovery was uneventful, and during the postoperative period no signs of discomfort or infection were noticed. At 10 days after transplantation, the animals were euthanized, and ovarian tissues were assayed by BLI, by real-time qPCR for AMH mRNA levels, and by histology of follicle numbers and morphology.

### Slow freezing

Ovaries taken from the donor mice after euthanasia were either transplanted fresh (controls) or cryopreserved slowly using a modification of the method of Gosden *et al.* [16]. For cryopreservation, the ovaries were suspended in a cryoprotectant medium of human tubal fluid (HTF) medium supplemented with 10% fetal bovine serum (FBS) and 1.5 M mol/L dimethylsulfoxide (DMSO, Sigma-Aldrich, St Louis, MO, USA) on crushed ice. Each ovary was then drawn up into the middle of a 0.25 mL plastic freezing straw with a small volume of cryoprotectant medium. The straws were sealed with polyvinyl chloride powder and held on ice for 30 min before they were placed in a planer programmable freezer precooled to 0°C. The straws were cooled at −2°C/min to −7°C and held for 5 min, seeded manually, held at −7°C for a further 10 min, cooled to −40°C at −0.3°C/min, further cooled to −140°C at −10°C/min and finally transferred to liquid nitrogen for storage. For thawing of the ovaries, the straws were removed from liquid nitrogen, held in air for 20 s and transferred to a water bath at room temperature for 10–20 s. The contents of the straws were emptied into HTF medium with 10% FBS. The DMSO was removed by repeated rinsing. Ovaries were kept in this medium at room temperature for 10 min before being used for transplantation.

### Bioluminescence imaging of transplanted ovaries

The Xenogen IVIS-50 imaging system (Xenogen, Hopkinton, MA, USA) was used for BLI. Mice were injected with 150 mg luciferin/kg (i.p.) 5 min before imaging. The luciferin solution was sterilized by passing it through a 0.22 μm filter. Mice were placed in the imaging chamber under continuous isoflurane (2%) anesthesia. The animals were rotated to the left to expose the flank optimally to the camera.

### Histology and follicle counting

Fixed fresh and frozen–thawed ovarian grafts were embedded in paraffin wax and serially sectioned at 6 μm, and the sections were stained with hematoxylin and eosin (HE) after dewaxing. Sections for analysis were taken at 36 μm intervals, and only follicles containing an oocyte were counted to avoid counting follicles twice. Healthy, nonatretic follicles were classified as primordial, primary, secondary, or antral. Primordial follicles had one layer of flattened granulosa cells surrounding the oocyte; primary follicles had a single layer to multiple layers of cuboidal granulosa cells without theca cells; secondary follicles had multiple layers of cuboidal granulosa cells, presence of theca cells and larger size than primary follicles; and antral follicles had a fluid-filled cavity adjacent to the oocyte.

### Reverse transcription (rt) and real-time qpcr

Single-strand cDNA was reverse transcribed from total RNA (2 μg) using a High Capacity cDNA Reverse Transcription kit (Applied Biosystems Pty Ltd.; Life Technologies Ltd.**,** Paisley UK) and random primers. The RT conditions consisted of 10 min of annealing at 25°C, 120 min of cDNA synthesis at 37°C and 5 min of inactivation at 85°C.

Genes for AMH and glyceraldehyde 3-phosphate dehydrogenase **(**GAPDH) were quantified by real-time qPCR with a StepOne thermal cycler (Applied Biosystems) using a commercial kit (Power SYBR Green PCR Master Mix; Applied Biosystems). The primers were designed using Primer-3 (http://primer3.ut.ee/) based on sequences in the GenBank database (http://www.ncbi.nlm.nih.gov). The amplification program consisted of an initial 10 min activation at 95°C followed by 40 cycles of PCR (each cycle consisting of 15 s of denaturation at 95°C and 60 s of annealing at 60°C). The amount of template cDNA used in each reaction was normalized to the amount of GAPDH mRNA. Amplification of template cDNA was in the linear range for the number of PCR cycles, and RT–PCR of GAPDH transcripts was performed using the same amount of cDNA from each sample as a template. The mRNA levels were normalized to those of GAPDH mRNA and were expressed as a percentage of the mRNA levels of fresh ovaries transplanted for 1 day, which was taken as 100%. Oligonucleotide PCR primers were selected with the assistance of a computer program (Primer-3) designed to optimize GC contents and melting temperature, and to minimize hairpin and dimer formation. The selected PCR primers included the following:

- GAPDH forward, 5′–ACCCAGAAGACTGTGGATGG–3′;
- GAPDH reverse, 5′–TGTGAGGGAGATGCTCAGTG–3′;
- AMH forward, 5′–CTATTTGGTGCTAACCGTGGACTT–3′;
- AMH reverse, 5′–AAGGCTTGCAGCTGATCGAT–3′.

### Statistical analysis

All data including follicle counts and the relative mRNA levels of molecular marker expression are expressed as the mean ± SD and were analyzed by univariate analysis of variance (ANOVA). All analyses were conducted using the statistical software package R version 2.6.2 (R Foundation for Statistical Computing, Vienna, Austria), and *P* < 0.05 was considered statistically significant.

## Results and discussion

**Figure 2:**
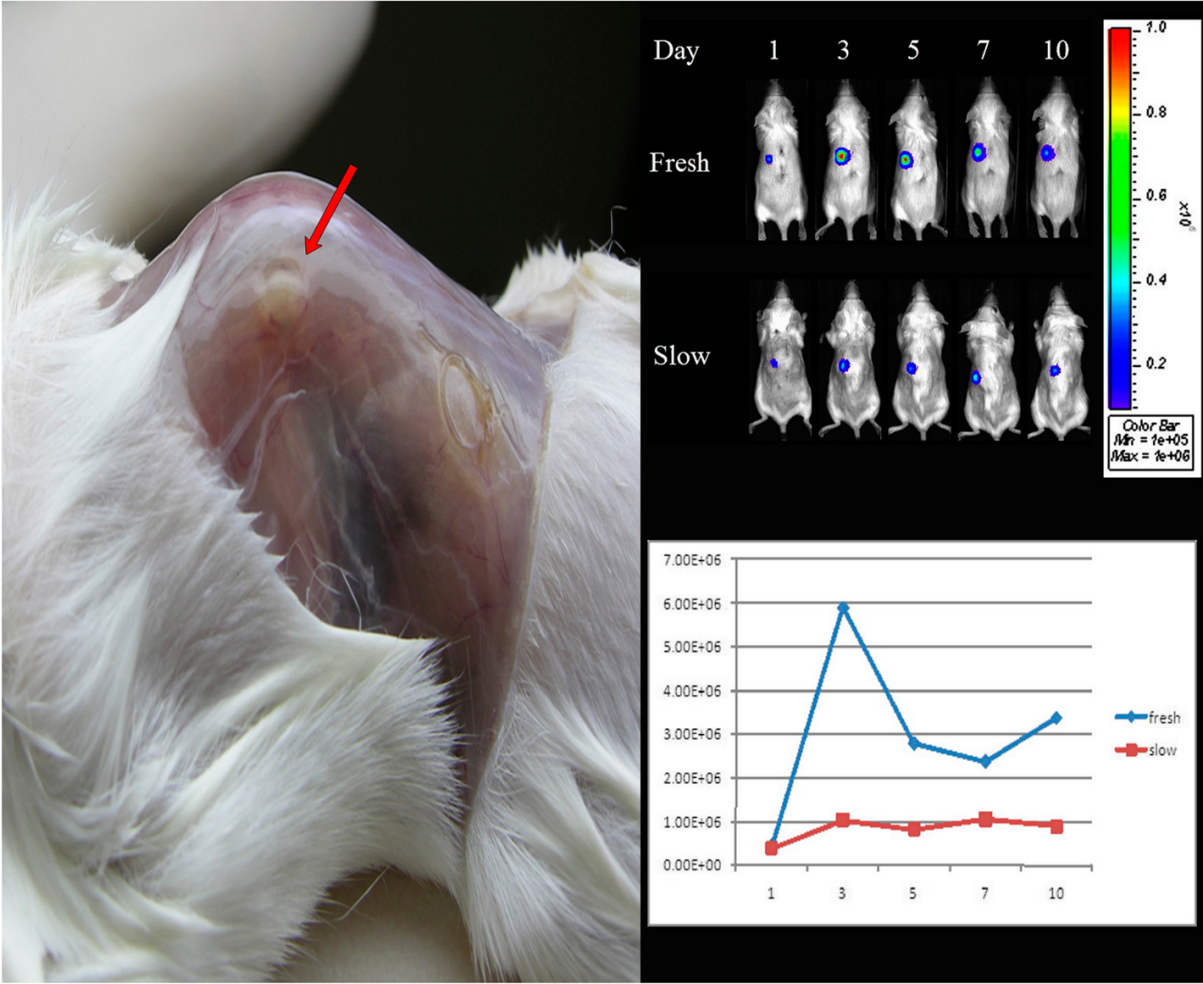
**Ovaries of a transgenic FVB/N-Tg** ***(PolII–Luc)*** **Ltc mouse were transplanted under the back muscle (arrow) of FVB/N wild-type recipient mouse (left).** *In vivo* BLI of mouse ovarian grafts in fresh and frozen–thawed ovary transplant groups on days 1, 3, 5, 7 and 10 after transplantation (upper right). In the fresh ovaries, bioluminescence increased initially on day 3, then decreased between days 3 and 7 and rose again from day 7 to day 10 with a subsequent cyclic fluctuation in intensity. In the frozen–thawed ovaries, the bioluminescence was no different after transplantation (lower right).

There were significantly stronger BLI signals in the transplanted fresh control ovaries than in the frozen–thawed ovaries. In the fresh ovaries, bioluminescence increased initially on day 3, decreased between days 3 and 7 and rose again from day 7 to day 10 with a subsequent cyclic fluctuation in intensity. However, there was measurable BLI output and cyclic fluctuation up to day 10 after transplantation in the frozen–thawed ovaries (Figure 2).

**Figure 3:**
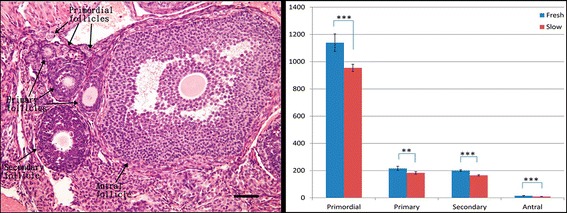
**Follicles were classified as primordial, primary, secondary, or antral (arrow).** Primordial follicles had one layer of flattened granulosa cells surrounding the oocyte; primary follicles had a single layer to multiple layers of cuboidal granulosa cells without theca cells; secondary follicles had multiple layers of cuboidal granulosa cells, presence of theca cells and larger size than primary follicles; and antral follicles had a fluid-filled cavity adjacent to the oocyte. Bar = 50 μm (Left). Estimated primordial, primary, secondary and antral follicle counts of fresh and frozen–thawed ovaries on day 10 after transplantation. The total numbers of follicles in frozen–thawed ovaries were significantly decreased compared with the transplanted fresh controls. Data are expressed as the mean ± standard deviation. ***P < 0.001(Right).

**Figure 4:**
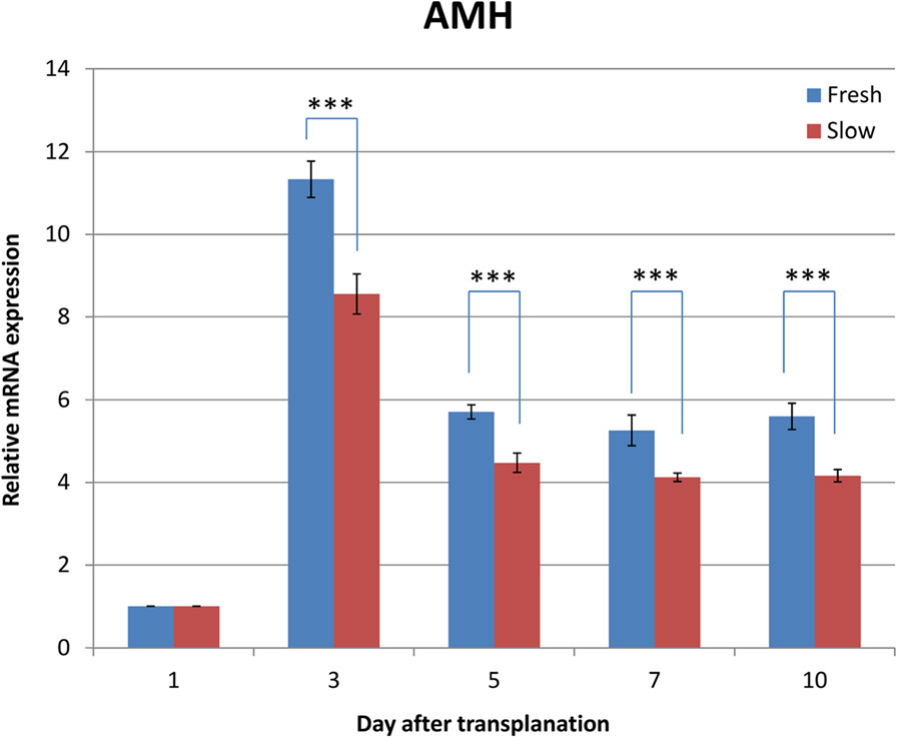
**The AMH mRNA expression in fresh and frozen–thawed transplanted ovaries over time (days 1, 3, 5, 7 and 10).** The mRNA expression in fresh and frozen–thawed ovaries increased on day 3 after transplantation and declined gradually thereafter. Data are expressed as the mean ± standard deviation. ***P < 0.001.

In order to determine the effect of cryopreservation of ovarian tissues, the number of primordial, primary, secondary and antral follicles was counted in ovarian grafts 10 days after transplantation (Figure 3). The total numbers of follicles in frozen–thawed ovaries were significantly decreased compared with fresh controls (*P* < 0.001). In addition, we evaluated the level of AMH mRNA in fresh and cryopreserved ovaries after transplantation (Figure 4). The AMH mRNA expression in both groups declined on day 3 after transplantation. The level of AMH was significantly higher in the fresh ovaries than those frozen–thawed (*P* < 0.001), showing that the latter ovaries had suffered unavoidable harm after transplantation.

The number of primordial follicles was significantly lower in frozen–thawed ovaries compared with fresh controls at 10 days after transplantation. The mRNA expression of AMH was also significantly higher in fresh ovarian grafts than in the frozen–thawed ones. The reduced mRNA level and primordial follicle counts in the transplanted frozen–thawed ovaries can be attributed to cryoinjury and subsequent ischemia after transplantation. In general, the aim of cryopreservation of ovarian tissue is to isolate the ovarian cortex from the medulla to reduce the thickness of the transplant, thereby optimizing follicular survival [17].

The major challenges for ovarian cryopreservation include defining an optimal protocol for cryopreserving large tissue masses, development of nontoxic cryoprotectants and prevention of ischemia–reperfusion tissue damage. The follicular survival rate obtained with frozen–thawed human ovarian tissue is approximately 70%–80% [7,17]. Choi *et al*. [18] evaluated the effect of cryopreservation (slow freezing and vitrification) on the development of frozen–thawed mouse primordial follicles. A significant reduction was noted in the developmental rate after *in vitro* culture of primordial follicles derived from both types of freezing methods, with a slight reduction compared with controls after 5 days of culture. Earlier studies revealed that almost 50% of primordial follicles are lost because of initial ischemia rather than during the freeze–thaw process itself [19–21].

Although primordial follicles might be resistant to freezing–thawing [7,17], the damage may be contributed by subsequent transplantation without vascular anastomosis [22]. Tissue ischemia without vascular anastomosis remains a problem for implants because the process of revascularization takes 2–7 days to complete, depending on the size of the implant [16,23]. Donnez *et al*. [24] stated that the transplantation of ovarian tissue pieces without a vascular pedicle requires the establishment of a new blood supply that takes 5 days. This leads to a substantial loss of follicles because of hypoxia and ischemia of the graft. Van Eyck *et al*. [25,26] found that human ovarian xenografts were exposed to hypoxia before day 5 and were progressively revascularized by day 10. In order to evaluate these short- and long-term tissue variations, BLI may be used as a convenient noninvasive technique for following early or late whole tissue changes *in vivo*.

Bioluminescence imaging is a nondestructive technique capable of repeated measurements [27]. Its advantages include sensitivity, efficiency, relatively low cost and versatility. Most imaging systems provide 2-dimensional (2D) information in small animal models, showing the locations and intensity of light emitted from the animal in pseudocolor scaling. A 3-dimensional (3D) capability for BLI is now available but is more expensive and less efficient. This technique is now being used to detect the location and burden of xenografted tumors, or to identify and measure the number of immune or stem cells after adoptive transfer. Specialized applications of BLI also follow tissue-specific luciferase expression in transgenic mice and monitoring of biological processes such as signaling or protein interactions in real time [15]. Here, the application of 2D BLI was the first attempt to investigate cryopreserved ovarian grafts *in vivo* over time. It has shown that we could use BLI to explore optimal protocols for ovarian cryopreservation.

In our previous studies, we applied BLI as a tool to evaluate the effects of immunosuppression after allotransplantation of ovarian grafts [28], to track the rejection and survival of mouse ovarian iso- and allografts, and to evaluate germ cells *in vitro* and transplantation *in vivo* as a part of fertility preservation in prepubertal male mice [29,30].

Folliculogenesis in ovarian grafts has been evaluated by molecular biology and histology. An ovary-specific gene such as that for growth differentiation factor-9 (GDF-9) might serve as a landmark for studying folliculogenesis. In our study, the Pol II promoter was used to evaluate the tissue *in situ* using BLI. Most such imaging systems provide 2D information, showing the locations and intensity of light emitted from the animal in pseudocolor scaling. In this, the algorithm tries to reconstruct what is happening inside the mouse by providing an approximate 3D reconstruction. Then, a digital mouse atlas is overlaid onto the 3D diffuse tomography reconstruction to obtain anatomical reference points. The Caliper algorithm for 3D reconstruction takes longer because it is based on the assumption that the mouse is optically homogeneous. Hence, 3D diffuse tomography reconstruction seems to perform well for a parenchymal organs like the liver.

The drawback of our study is that the Pol II promoter may only be able to quantify viable tissue of the ovarian graft, and not each stage of folliculogenesis individually. Therefore, it will be essential to design ovary-specific promoters for following the fate of each stage of folliculogenesis after protocols using cryopreservation and different transplantation sites. In brief, we have paved the way to demonstrating a model of *in vivo* imaging by BLI for ovarian graft transformation and survival. The impact of these interventions on folliculogenesis needs to be evaluated by versatile study designs.

## Conclusions

Our study suggests that ovarian cryopreservation using slow freezing compromised the ovarian reserve at an early stage after transplantation. This was caused by cryoinjury and ischemia leading to follicle loss. This study also demonstrates the promise of luciferase gene-based systems, which are widely used in the field of genetic engineering as reporters. Analysis by *in vivo* BLI has also been harnessed for reproductive biomedical research and minimizes biological variation. A noninvasive *in vivo* approach does not require sacrificing the experimental animal and thereby reduces the numbers required for experimentation because multiple measurements can be made in the same animal over time.

## Abbreviations

BLI: *In vivo* bioluminescent imaging
AMH: Anti-Müllerian hormone
qPCR: Quantitative polymerase chain reaction
Pol: Polymerase
i.p.: Intraperitoneally
HTF: Human tubal fluid
FBS: Fetal bovine serum
DMSO: Dimethylsulfoxide
HE: Hematoxylin and eosin
RT: Reverse transcription
GAPDH: Glyceraldehyde 3-phosphate dehydrogenase
3D: 3-dimensional
SD: Standard deviation
